# University of Michigan

**Published:** 1864-04

**Authors:** 


University of Michigan.—It is stated in the printed catalogue of this institution, that the number of students in the Medical Department at the last session was 340.

